# A case of iliopsoas hematoma as a complication of tetanus in a patient who did not receive anticoagulant therapy

**DOI:** 10.1186/s12879-020-05455-z

**Published:** 2020-10-07

**Authors:** Hiroki Nagasawa, Ken-ichi Muramatsu, Ikuto Takeuchi, Yoshihiro Kushida, Kei Jitsuiki, Jun Shitara, Hiromichi Ohsaka, Kazuhiko Omori, Yasumasa Oode, Youichi Yanagawa

**Affiliations:** Department of Acute Critical Care Medicine, Shizuoka Hospital, Juntendo University, 1129 Nagaoka, Izunokuni City, Shizuoka 410-2295 Japan

**Keywords:** Tetanus, Iliopsoas hematoma, Complication, Anticoagulation therapy

## Abstract

**Background:**

The specific clinical feature of tetanus is whole body muscle spasms. These spasms are intensely painful and sometime lead to some injuries. Vertebral fractures have been reported as a common complication of tetanus, however iliopsoas hematoma is a rare complication. We describe a case of iliopsoas hematoma in a tetanus patient who had not been treated with any anticoagulant or antiplatelet agents.

**Case presentation:**

A 72-year-old female patient was transferred to our hospital 7 days after the onset of tetanus. An iliopsoas hematoma was identified in her right iliopsoas muscle on computed tomography. There was no extravasation; thus, the hematoma improved with conservative therapy. There were no episodes that suggested a bleeding tendency, or no factors associated with hemorrhagic conditions.

**Conclusion:**

This is the first report of iliopsoas hematoma as a complication in a tetanus patient who did not received anticoagulation therapy. The possibility of IPH as a complication of tetanus should be considered before and during the administration of anticoagulation therapy.

## Background

Tetanus, a disease that has killed many people since ancient times is caused by tetanospasmin, a toxin produced by *Clostridium tetani*, which is widely distributed in soil and the human intestines. On the other hand, the neurological prognosis is relatively good. The specific clinical feature of tetanus is whole body muscle spasms (e.g., lock jaw and opisthotonus); these spasms are intensely painful and sometime lead to bone fracture [1].

Common complications of tetanus include hospital-acquired pneumonia (HAP), ventilator-associated pneumonia (VAP), infections (e.g., sepsis), deep vein thrombosis (DVT), pulmonary thromboembolism (PTE) and upper gastrointestinal hemorrhage [1, 2]. Vertebral fractures have previously been reported as a complication of opisthotonus [1, 3, 4]. However, iliopsoas hematoma (IPH) is a rare complication of tetanus, with only one report of IPH in a tetanus patient who was treated with anticoagulant therapy to prevent DVT/PTE [5]. We herein describe a case of iliopsoas hematoma in a tetanus patient who had not been treated with any anticoagulant or antiplatelet agents.

## Case presentation

A 72-year-old female patient, who was not prescribed any medications, without any relevant medical history, was transported to our hospital due to suspected tetanus. She had never been vaccinated against tetanus. Fourteen days previously, she had been injured on her right forearm by a rose thorn while gardening. She did not visit any hospital, and took treated her injury herself. On the 10th day after injury (day 1), trismus suddenly appeared. Two days later (day 3) she presented to another hospital with a chief complaint of lock jaw and anorexia, and she was hospitalized for an unknown disease. The muscle tone of her limbs gradually increased after admission, and opisthotonus appeared on days 5–6. Tetanus was suspected, and she was transported to our hospital on day 7.

**Fig. 1:**
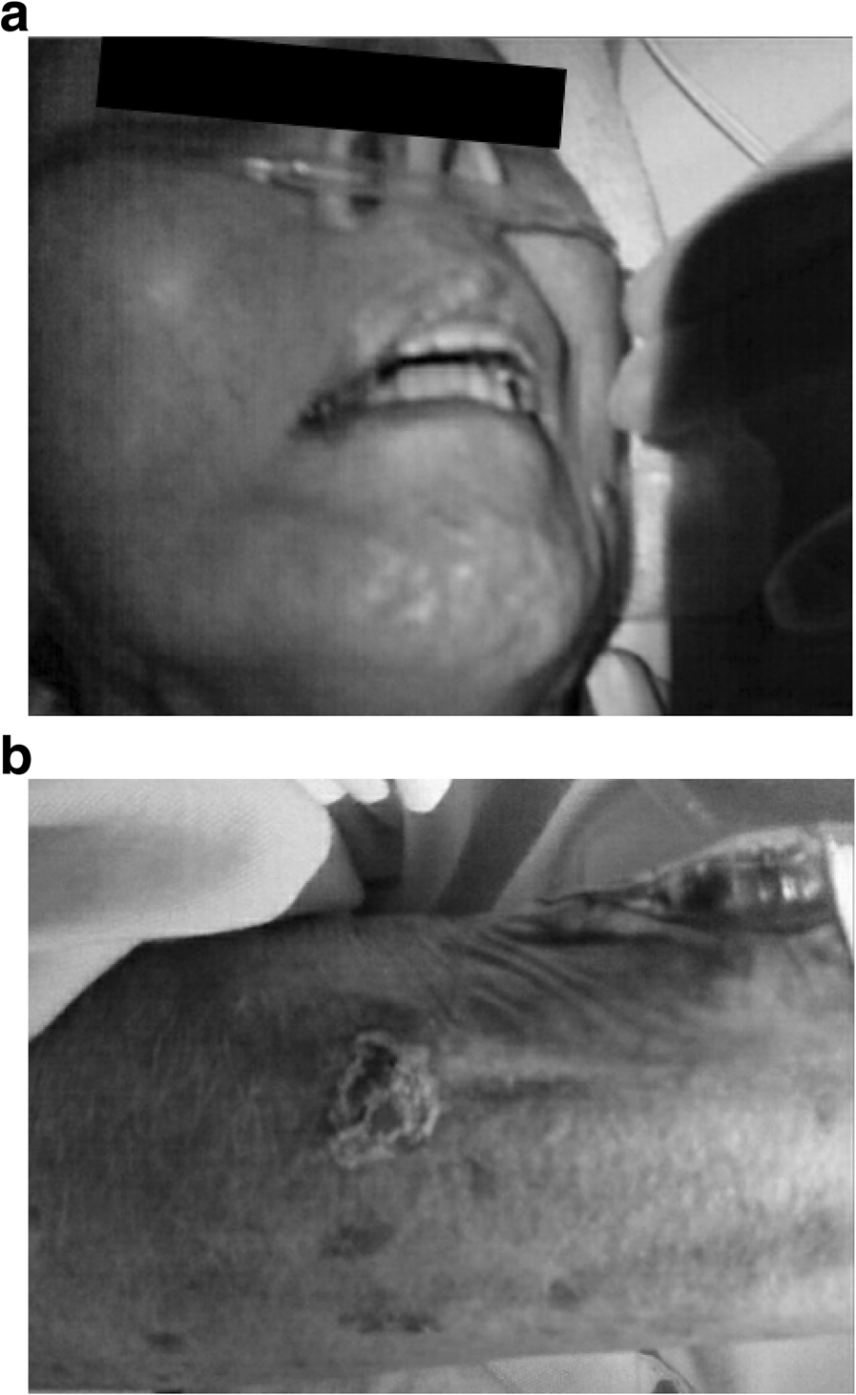
**a** A 77-year-old woman with tetanus. Her mouth could not open no more than two finger breadths. **b** The patient’s right forearm was injured by a rose thorn. This wound, which was already covered with scabs, seemed to be the focus of tetanus infection

On general examination when she arrived at our emergency department, her consciousness was alert. Her vital signs were as follows: blood pressure, 128/88 mmHg; heart rate, 82 beats/minute, respiratory rate, 24 breaths/minute, and body temperature, 37.9 °C. A physiological examination revealed trismus as small as 2 finger breadths (Fig. 1a), dysphagia, and limb muscle spasm or spinal stiffness that were triggered by stimuli such as light, sounds and a change of body position. The wound on her right forearm was covered by a scab and no debridement was needed (Fig. 1b).

**Fig. 2:**
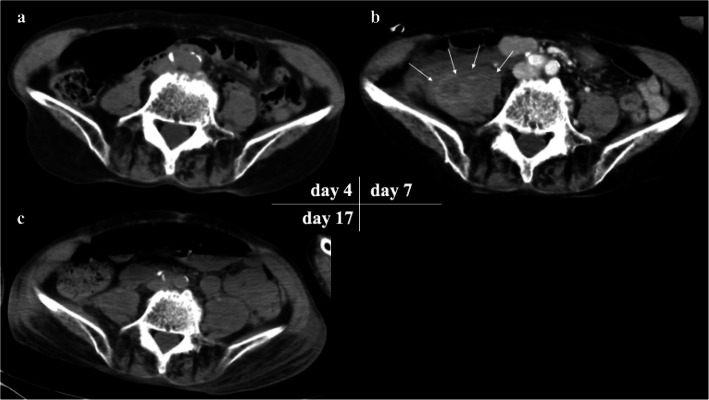
There was no hematoma on day 4 (**a**). Right iliopsoas hematoma was unexpectedly found without extravasation on day 7. White arrows (↓) indicate hematoma in the right iliopsoas muscle (**b**). After conservative treatment, the iliopsoas hematoma become unclear on day 17 (**c**)

Whole body computed tomography (CT) was performed on admission and revealed a hematoma of the right iliopsoas muscles without extravasation (Fig. 2b). No hematoma was observed on CT images from a scan performed on day 4 (Fig. 2a). We decided to perform conservative therapy for IPH.

The patient transferred to the intensive care unit (ICU). She was sedated with diazepam and intubated, then mechanical ventilator management was started because she complained of painful spasms. Human tetanus immune globulin (3000 units) was administered intravenously. Penicillin G (8,000,000 units/day) and Ceftriaxone (2 g/day) were administered to treat local infection and pneumonia, which was found on CT after her admission. The continuous administration of midazolam and magnesium sulfate hydrate (50–75 g/day) was started, and a Kampo medicine, shakuyakukanzoto (7.5 g/day), was administered by nasogastric tube to relieve the pain caused by her muscle spasms. Muscle relaxants, such as rocuronium and vecuronium, were not required during her hospital stay.

On day 10, we performed tracheostomy in anticipation of the need for long-term mechanical ventilation. On day 17, a CT scan confirmed that the IPH had been absorbed (Fig. 2c), and we started heparin calcium (10,000 units/day) with subcutaneous injection for the prevention of DVT, where whole-body muscle spasm subsided around day 21, then the mechanical ventilator became dislodged. She was transferred from the ICU to the general medical ward on day 24. During her hospital day, she developed some complications, including HAP/VAP, catheter-associated infection, methicillin-resistant *Staphylococcus aureus* pneumonia, cardiopulmonary arrest due to obstruction by sputum; however, all of complications were overcome. The patient underwent rehabilitation in our hospital and was discharged to her home on the day 95.

Prior to the patient’s hospitalization, there were no episodes that suggested a bleeding tendency. We performed blood tests to check for any factors associated with hemorrhagic conditions, such as hemophilia; all of these tests were within the normal limits.

## Discussion and conclusion

Tetanospasmin is transferred from the peripheral nerves to the central nervous system, where it inhibits release of glycine and gamma-aminobutyric acid (GABA) and affects the inhibitory motor neurons and autonomic nervous system [1, 2, 6]. These neurons disorders lead to complications such as autonomic dysfunction, arrhythmia, takotsubo cardiomyopathy, cardiac arrest, respiratory failure, ileus, diarrhea and renal failure [1, 2, 7]. In some previous reports vertebral fractures was reported in patients with severe opithotonus [1, 3, 4].

Ishii et al. reported that 4 of 21 (19%) patients with tetanus developed iliopsoas hematoma as a complication during their clinical course [5]. Iliopsoas hematoma is reported to occur in 0.3% of ICU patients [8, 9]; thus, the rate of 19% seems high. No reports have suggested a relationship between tetanus and IPH, and this is the first report to demonstrate that IPH may be a complication of tetanus. In the report by Ishii et al., all 4 cases with IPH received anticoagulation therapy. Thus, it was not possible to determine whether tetanus or anticoagulation therapy was the main cause of IPH [5].

Commonly reported risk factors for IPH include trauma, anticoagulation therapy, and hemodialysis [8–10]. In our report, patient, CT scans obtained on day 4, when opisthotonus appeared, showed no sign of IPH; however, hemorrhage was observed in the right iliopsoas muscle on day 8, before the administration of heparin. This patient’s body had not been injured and she was not a dialysis patient. Furthermore, we did not detect any underlying disease associated with a bleeding tendency. From these results, we diagnosed the IPH as a complication of tetanus.

The mortality rate of IPH in the ICU has been reported to be 30–50% [8, 9]. On the other hand, in all five of the reported cases of IPH with tetanus, including those reported by Ishii et al. [5], the patient survived; thus, IPH with tetanus may not affect the outcomes.

In the management of severe tetanus, sedation and muscle relaxants are routinely required; thus, it may be difficult to notice the patient’s complaint. If our patient had shown back pain, we might incorrectly have concluded that it had been caused by opisthotonus. IPH was found by chance in the present case, without any factors suggestive of bleeding (e.g., hypotension or decreased hemoglobin). Thus, physicians should pay attention to the possible occurrence of IPH in tetanus patients before or during the administration of anticoagulation therapy.

In Japan, penicillin is still the first choice for antibiotics to treat tetanus [11], and some reports have described the use of penicillin in the treatment of tetanus [7, 12]. Metronidazole infusion was introduced in Japan in 2014; however, its use in tetanus treatment is not covered by insurance. One report noted that the doses of muscle relaxants and sedative required by tetanus patients were lower in those who received metronidazole [13]. In the present case, we conventionally selected penicillin, however, the duration of her illness might have been improved if she was treated with metronidazole instead of penicillin.

In Japan, the incidence of tetanus among young people has decreased since the introduction of the diphtheria-tetanus-pertussis (DTP) vaccine; however, tetanus morbidity remains a problem among patients > 60 years of age [14]. It should be kept in mind that “prevention” — in particular booster vaccination in adults —is of the utmost importance in the management of tetanus.

This is the first report to describe IPH as a complication of tetanus in a patient who was not receiving anticoagulation therapy. It is important to prevent thrombosis in tetanus patients; however, the possibility of IPH as a complication of tetanus should be considered before and during the administration of anticoagulation therapy.

## Data Availability

The datasets used during the current study are available from the corresponding author on reasonable request.

## Abbreviations

CT: Computed tomography
DTP: Diphtheria-tetanus-pertussis
DVT: Deep vein thrombosis
GABA: Glycine and gamma-aminobutyric acid
HAP: Hospital-acquired pneumonia
ICU: Intensive care unit
IPH: Iliopsoas hematoma
PTE: Pulmonary thromboembolism
VAP: Ventilator-associated pneumonia
